# Nonenzymatic DNA-Based Fluorescence Biosensor Combining Carbon Dots and Graphene Oxide with Target-Induced DNA Strand Displacement for microRNA Detection

**DOI:** 10.3390/nano11102608

**Published:** 2021-10-03

**Authors:** Yuanyuan Gao, Hong Yu, Jingjing Tian, Botao Xiao

**Affiliations:** 1 Guangdong Provincial Key Laboratory of Fermentation and Enzyme Engineering, School of Biology and Biological Engineering, South China University of Technology, Guangzhou 510006, China; 202010108508@mail.scut.edu.cn (Y.G.); 201920146464@mail.scut.edu.cn (H.Y.); 2 State Key Laboratory of Marine Resource Utilization in South China Sea, School of Material Science and Engineering, Hainan University, Haikou 570228, China; tianjingjinghubei@163.com; 3 Key Laboratory of Emergency and Trauma of Ministry of Education & Research Unit of Island Emergency Medicine of Chinese Academy of Medical Sciences, Hainan Medical University, Haikou 571199, China; 4 Joint International Research Laboratory of Synthetic Biology and Medicine, School of Biology and Biological Engineering, South China University of Technology, Guangzhou 510006, China

**Keywords:** carbon dots, graphene oxide, microRNA, fluorescence resonance energy transfer, toehold-mediated strand displacement reactions

## Abstract

Based on a fluorescence “on-off-on” strategy, we fabricated a simple and highly sensitive DNA-based fluorescence biosensor for the detection of micro (mi)RNA from carbon dots (CDs) and graphene oxide (GO) without complicated and time-consuming operations. CDs were successfully synthesized and conjugated to the end of a single-stranded fuel DNA that was adsorbed onto the surface of GO through π-π stacking, resulting in fluorescence quenching. In the presence of the target miRNA let-7a, the fuel DNA was desorbed from the GO surface, and fluorescence was restored through two successive toehold-mediated strand displacement reactions on double-stranded DNA-modified gold nanoparticles. The target miRNA let-7a was recycled, leading to signal amplification. The concentration of let-7a was proportional to the degree of fluorescence recovery. Under optimal conditions, there was a good linear relationship between the relative fluorescence intensity and let-7a concentration in the range of 0.01–1 nM, with a detection limit of 7.8 pM. With its advantages of signal amplification and high biocompatibility, this fluorescence sensing strategy can be applied to the detection of a variety of target miRNAs and can guide the design of novel biosensors with improved properties.

## 1. Introduction

Micro (mi)RNAs are a type of noncoding single-stranded small RNA with a length of about 20–24 nucleotides encoded by endogenous genes [1] that participate in the regulation of normal cellular activities, including proliferation, differentiation, and apoptosis, among others [2]. MiRNAs show dysregulated expression in many cancers and have both tumor suppressor and proto-oncogenic functions [3]. The miRNA let-7a is a tumor suppressor [4,5] whose elevated expression during the differentiation of neural stem cells prevents the death of neurons in the absence of growth factors [6]. Sensitive methods for the detection of cancer-related miRNAs are important for early diagnosis as well as for monitoring disease progression and treatment response [7]. However, the characteristics of miRNAs, including a small size [8], low abundance, and high degree of sequence homology among family members, make their detection technically challenging [9]. A variety of approaches are used to detect miRNAs, including quantitative real-time reverse transcription PCR [10,11,12,13], northern blotting [14], DNA microarray analysis [15], electrochemical methods [16,17,18], and nanomaterial-assisted amplification technology [19,20,21]. Although they can detect miRNAs with high sensitivity, these methods have certain disadvantages. For example, they are often complicated [22], time-consuming, and require expensive equipment [23].

Carbon dots (CDs), along with fullerenes, carbon nanotubes, and graphene [24], are a type of nanomaterial with an approximately spherical zero-dimensional shape and a diameter <10 nm [25]. Owing to their stable optical properties, good water solubility, and multiple sources [26,27,28,29], CDs are widely used for metal ion [30,31,32,33] and protein [34] detection. CDs with different fluorescence yields and luminescence characteristics can be obtained through specific surface modifications [35,36]. Compared to traditional organic dyes and quantum dots based on heavy metals, CDs have many advantages such as a simple preparation method, low toxicity, and high biocompatibility and fluorescence yield. Thus, CDs have potential applications as fluorescent labels in bioimaging and sensing [37], are also used as drug carriers, and are promising nanomaterials for disease detection [28].

Graphene oxide (GO) is a two-dimensional carbon material with a thickness of 1 atom [38] that has excellent electrical conductivity, high water solubility, and a large specific surface area [39]. Due to its two-dimensional planar conjugated structure, GO has a higher quenching efficiency and shorter quenching time than some traditional quenchers [40] and can effectively quench the fluorescence of organic dye molecules and some quantum dots [41]. Single-stranded DNA has exposed bases and can be adsorbed onto the surface of GO through π-π stacking [42]; therefore, this interaction is a weak noncovalent force similar to hydrogen bonding or solvophobic and ion-dipole interactions that typically exist in or between molecules of compounds with a conjugated structure [43,44]. With its advantages of a high quenching efficiency and a low background signal [45], GO can be combined with specific probes to detect metal ions [46,47], enzyme activity [48], and nucleic acid molecules [49].

Gold nanoparticles (AuNPs) have a diameter between 0.5–250 nm and are usually in the form of a hydrosol dispersed in an aqueous solvent [50]. AuNPs are used in biosensors because of their high stability and can be easily conjugated to biomarkers through covalent bonds or noncovalent electrostatic interactions [51]. Additionally, AuNPs have a large specific surface area and can thus serve as a carrier [52] of multiple probes simultaneously, providing signal amplification and improving the sensitivity of the sensor [53]. DNA-AuNPs formed by conjugating DNA to the surface of AuNPs have excellent physical and chemical properties, including unique optical properties, biological recognition, antienzyme activity, and good biocompatibility [54,55]. Modifying AuNPs with sulfhydryl (–HS) DNA not only makes them more stable in salt solutions and minimizes agglomeration but also improves the stability of the entire sensing system [56], which is highly useful for diagnostic applications and nanostructure construction [57,58].

Toehold-mediated strand displacement reactions (TSDRs) are reactions in which one strand of dsDNA is replaced by another single strand by binding to a short single-stranded neighboring sequence (toehold) [59,60]. By predicting the thermodynamics and kinetics of the reaction, the required DNA sequence in the reaction is precisely designed [61] so that it can complete the displacement reaction without enzymes at room temperature [62,63].

In the present study, a fluorescence biosensor for detecting the miRNA let-7a was developed based on CDs-labeled fuel DNA (fuel DNA-CDs) and GO, with the former acting as an energy donor and molecule recognition probe and the latter as a fluorescence resonance energy transfer (FRET) acceptor and fluorescence quencher. In the absence of let-7a, single-stranded fuel DNA-CDs with exposed bases were adsorbed onto the surface of GO, and the fluorescence of CDs was quenched. In the presence of let-7a, two successive TSDRs were triggered, with the fuel DNA forming a duplex structure (dsDNA-AuNPs) that caused the fuel DNA-CDs to detach from the GO surface, resulting in fluorescence recovery. Let-7a was recycled during this process, which further amplified the signal. The concentration of let-7a was proportional to the fluorescence recovery value, making the sensor a useful tool for accurate target quantification. We achieved the sensitive and selective detection of let-7a in clinical serum samples based on this fluorescence “on–off–on” strategy.

## 2. Materials and Methods

### 2.1. Reagents

GO and AuNPs with a diameter of 15 nm were purchased from XFNANO Materials Tech Co., Ltd (Nanjing, China). N-Hydroxysuccinimide (NHS), 1-ethyl-3-(3-dimethylaminopropyl) carbodiimide (EDC), and Tris (2-carboxyethyl) phosphine hydrochloride (TCEP) were from Macklin Biochemical Technology Co., Ltd (Shanghai, China). Citric acid, ethylenediamine, Na_2_HPO_4_, NaH_2_PO_4_, NaCl, and KCl were purchased from Sinopharm Chemical Reagents Co., Ltd (Shanghai, China). Dialysis bags (3500 and 8000 MW) were purchased from Solarbio Science & Technology Co., Ltd (Beijing, China). Human serum samples were obtained from the First Affiliated Hospital of Hainan Medical University. All subjects gave their informed consent for inclusion before they participated in the study. The study was conducted in accordance with the Declaration of Helsinki, and the protocol was approved by the Ethics Committee of Hainan Medical University, China (No. HYLL-2018030). All oligonucleotides used in this study were high-performance liquid chromatography grade and were synthesized by Shanghai Sangon Biotechnology Co., Ltd (Shanghai, China). The sequences are listed in Table 1.

### 2.2. Apparatus

Fluorescence spectra were recorded on an FL-8500 fluorescence spectrophotometer (PerkinElmer, Shelton, CT, USA). Ultraviolet-visible (UV-vis) absorption spectra were recorded using a UV-2600 spectrophotometer (Shimadzu, Tokyo, Japan). CDs ultrastructure was examined by Talos L120C transmission electron microscopy (Thermo Scientific, Waltham, MA, USA). Fourier transform infrared (FT-IR) spectra were obtained under a transmission mode with a Tensor 27 spectrometer (Bruker Optik GmbH, Ettlingen, Germany).

### 2.3. Preparation of GO Solution

The GO solution was subjected to ultrasonic dispersion. Briefly, GO powder (10 mg) was added to deionized water (10 mL), and the mixture was immediately ultrasonicated in an ice bath for 3 h. A homogeneous brownish-yellow GO dispersion was collected and stored at room temperature until further use.

### 2.4. Preparation of CDs

Citric acid (3.0 g) and ethylenediamine (1875 μL) were added to distilled water (30 mL). The solution was transferred to a poly (tetrafluoroethylene) Teflon-lined autoclave (50 mL) and heated at 150 °C for 5 h. The solution was dialyzed with double-distilled water (molecular weight cutoff: 3500 Da); then, the prepared CDs (50.0 mg) were dispersed in an aqueous solution (50 mL) containing NaOH (2.5 g) and ClCH_2_COONa (2.5 g), followed by ultrasonication for 3 h. The generated CD-COONa was neutralized with HCl and dialyzed to obtain CDs with surface carboxyl groups. The synthesis yield of the CDs was 9%, and the fluorescence quantum yield of the CDs was 16% (excitation: 365 nm, emission: 460 nm, quinine sulfate as the standard).

### 2.5. Preparation of Fuel DNA-CDs Conjugates

The prepared CDs were dissolved in 10 mM phosphate-buffered saline (PBS [pH 7.5]) to obtain a CDs solution of 2.0 mg mL^−1^. NHS (50 mM, 1 mL) and EDC (500 mM, 1 mL) were added to the CDs solution (1 mL), followed by ultrasonication for 2 h. Fuel DNA was added, and the solution was incubated at 4 °C for 24 h. Excess CDs that were not attached to the ends of the fuel DNA were removed by dialysis (molecular weight cutoff: 8000 Da).

### 2.6. Preparation of dsDNA-AuNPs

DsDNA-AuNPs were prepared according to a previously described method [55]. Briefly, recognition DNA (thiolated oligonucleotide) was reduced for 1 h with TCEP at a molar ratio of 1:100 to prevent the formation of disulfide bonds. The recognition DNA and hairpin (HP) DNA were mixed in PBS (137 mM NaCl, 10 mM phosphate, 2.7 mM KCl (pH 7.4)) at a molar ratio of 1:1.2. The mixture was heated to 75 °C for 10 min and allowed to slowly cool to room temperature. The mixture (3000 nM) was added to the AuNP solution (20 nM), which was maintained at room temperature overnight. The solution was centrifuged at 13,000× *g* rpm for 20 min and the supernatant was decanted to remove unbound DNA. The solution was washed twice and stored in PBS solution in the dark at 4 °C.

### 2.7. Let-7a Fluorescence Detection

The detection of the miRNA let-7a fluorescence was performed as follows: fuel DNA-CDs solution (50 nM) was added to the GO solution and reacted for 2 min to completely quench the fluorescence; then, dsDNA-AuNPs were added along with different concentrations of let-7a (0 pM–1 nM), followed by incubation at 37 °C for 25 min. The fluorescence of the mixture was recorded with a spectrophotometer at excitation and emission wavelengths of 488 and 543 nm, respectively.

## 3. Results and Discussion

### 3.1. Design of the Biosensor

**Figure 1:**
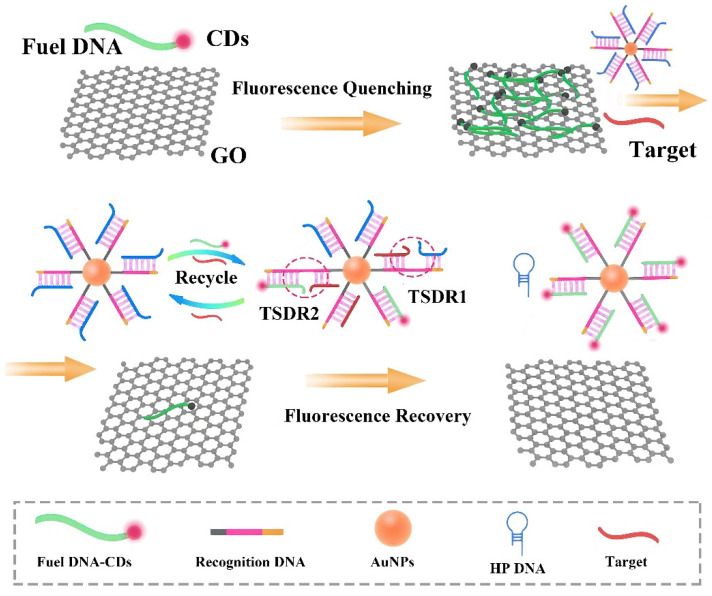
Schematic of nonenzymatic miRNA detection based on the CDs-labeled fluorescent probe and GO.

The underlying principles and steps for the detection of the target miRNA let-7a in a biological sample (human serum) are illustrated in Figure 1. Recognition DNA (containing gray, pink, and yellow sequences) first formed double-stranded (ds)DNA with HP DNA (blue sequence) through complementary base pairing. The end of the recognition DNA was modified with an –HS group bonded to the AuNPs through Au-S bonds, forming dsDNA-AuNPs. The two ends of the recognition DNA strand contained two special toehold regions (gray and yellow sequences) that triggered two sequential TSDRs; one end (gray sequence) bound to let-7a (red sequence) in the first reaction (TSDR1), and the second end (yellow sequence) combined with fuel DNA (green sequence) to trigger the second reaction (TSDR2). In the absence of let-7a, GO adsorbed single-stranded fuel DNA labeled with CDs through hydrophobic interactions and π-π stacking and completely quenched the fluorescence of CDs through FRET. In the presence of the target miRNA, let-7a triggered TSDR1, binding to the gray sequence of the recognition DNA and replacing the HP DNA. Additionally, HP DNA released during TSDR1 exposed the second toehold area, triggering TSDR2; the presence of the target was required for this reaction. Similarly, fuel DNA-CDs combined with the yellow sequence of the recognition DNA and displaced let-7a through toehold-mediated strand migration in TSDR2. Consequently, let-7a was recycled, the fuel DNA was desorbed from the GO surface, and the fluorescence of CDs was restored. The concentration of let-7a was directly proportional to the fluorescence recovery value.

### 3.2. Detection of Target miRNA by the Biosensor

The CDs were examined by Transmission Electron Microscopy (TEM), and the image was shown in Figure S1a, wherein the average particle size of the CDs was (11.6 ± 0.2) nm. The photos taken after the CDs solution was excited by green light at room temperature show that fluorescence will only be generated under the conditions in the specific wavelength range (Supplementary Materials, Figures S1b and S2). As shown in Figure S1c, the maximum excitation wavelength of CDs was 488 nm, and the corresponding emission wavelength was 543 nm. CDs were excited by blue light in the gel imaging system to emit fluorescence (Figure S3c), and this phenomenon was not observed under white light and UV light (Figure S3a,b). Figure S1d shows the FT-IR spectrum of the CDs, and the absorption bands at 2957 cm^−^^1^ and 1387 cm^−^^1^ were characteristic of –CH stretching vibration and bending vibration, respectively. The other absorption bands were assigned as follows: 1653 cm^−^^1^ and 1557 cm^−^^1^ for –C=O stretching vibration and 1050 cm^−^^1^ for –C–O stretching vibration, indicating that CDs have excellent water solubility due to the abundant oxygen-containing functional groups on the surface, which also enables stable photoluminescence performance and facile molecular modification with DNA. The UV absorption spectra and more detailed fluorescence spectra of CDs were shown in Figures S4 and S5. We also measured the fluorescence response of the CDs to other biologically relevant parameters, such as pH and ionic strength (Figure S6).

**Figure 2:**
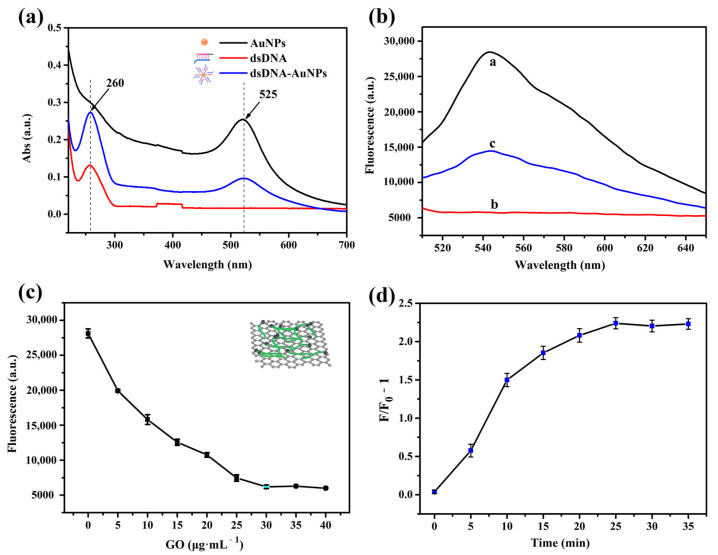
(**a**) Characterization of AuNPs, dsDNA, and dsDNA-AuNPs by UV–vis absorption spectroscopy. The concentration of AuNPs was slightly greater than that of dsDNA-AuNPs to make the difference between the curves obvious. (**b**) Fluorescence emission spectra of fuel DNA (50 nM) under different conditions: (**a**) fuel DNA-CDs in PBS; (**b**) fuel DNA-CDs + 30 μg/mL GO; (**c**) fuel DNA-CDs + 30 μg/mL GO + 1 nM let-7a in the presence of dsDNA-AuNPs (excitation: 488 nm, emission: 543 nm). (**c**) Fluorescence quenching of CDs labeled on the fuel DNA in the presence of GO at different concentrations. (**d**) Effects of the reaction time of two TSDRs on the *F/F_0_* −1 value of sensor system. Conditions: microRNA let-7a, 1 nM.

We examined whether the dsDNA formed by the hybridization of recognition DNA and HP DNA was conjugated to AuNPs to form a dsDNA-AuNP structure by UV–vis absorption spectroscopy. Unmodified AuNPs (i.e., without dsDNA) had a single absorption peak at 525 nm, whereas dsDNA without AuNPs had a single peak at 260 nm corresponding to electron interactions between bases in the DNA molecule (Figure 2a). DsDNA-AuNPs formed by the Au-S bond between dsDNA and AuNPs had two characteristic absorption peaks at 260 and 525 nm. The formation of the dsDNA-AuNPs structure was a prerequisite for the two sequential TSDRs in the presence of let-7a.

Next, we assessed the feasibility of detecting let-7a with the biosensor. Single-stranded fuel DNA-CDs were adsorbed to the GO surface through π-π stacking and hydrophobic interactions (Figure 2b), and the fluorescence of CDs was almost completely quenched by GO within about 2 min (curve b). After adding 1 nM let-7a, the TSDRs were initiated, causing fuel DNA-CDs to form double strands and desorb from the GO surface, resulting in fluorescence recovery (curve c) and demonstrating that the fluorescence signal of the sensor changes as a result of let-7a detection.

In the fluorescence quenching experiment, too high a GO concentration prevented fluorescence recovery after the addition of the target miRNA, whereas too low a concentration caused incomplete fluorescence quenching, which increased the background signal. We, therefore, optimized the concentration of GO to maximize fluorescence quenching. As the GO concentration in fuel DNA-CDs increased, the single-stranded fuel DNA was adsorbed onto the surface of GO, leading to FRET between CDs and GO and the quenching of fluorescence derived from CDs (Figure 2c). At 30 μg/mL GO, there was complete fluorescence quenching; therefore, this was taken as the optimal GO concentration and was used in subsequent experiments.

The experimental condition of the two TSDRs reaction time for the fluorescence assay was also optimized, as shown in Figure 2d. After adding the target miRNA let-7a, when the reaction time reaches 25 min, the fluorescence does not continue to recover, indicating that the two TSDRs reactions were complete. So we took the fluorescence value at 25 min after adding the target as the final fluorescence recovery value. We also explored and optimized other experimental conditions and finally took 30 nM dsDNA-AuNPs and 50 nM fuel DNA-CDs as the optimal experimental conditions for subsequent fluorescence recovery experiments (Figure S7).

### 3.3. Performance and Selectivity of the Biosensor

**Figure 3:**
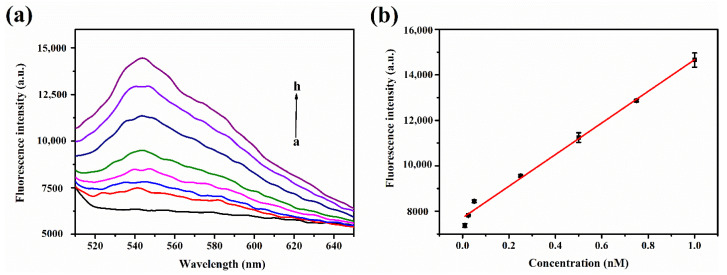
Performance of the biosensor as determined by fluorescence recovery in the presence of target miRNA (**a**) Fluorescence emission spectra of the dsDNA-AuNPs probe in response to various concentrations of the target miRNA let-7a. From a–h: 0, 0.01, 0.025, 0.05, 0.25, 0.5, 0.75, and 1 nM (excitation: 488 nm, emission: 543 nm). (**b**) Calibration curve showing the linear relationship between let-7a concentration and fluorescence intensity.

Fluorescence recovery gradually increased with let-7a concentration (Figure 3a). There was a good linear relationship between fluorescence intensity and let-7a concentration in the range of 0.01–1 nM (Figure 3b). The linear regression equation was *F* = 6955.58 × *c* + 7711.98 (where *F* is the fluorescence recovery value and *c* is the let-7a concentration), and the correlation coefficient was *R^2^* = 0.9868. The limit of detection (LOD) and limit of quantitation (LOQ) were calculated using the following formula: LOD = 3 *S_b_*/*S*, LOQ = 10 *S_b_*/*S* (where *S_b_* is the standard deviation of 11 blank measurements and *S* is the slope of the calibration curve). The final calculated value of LOD and LOQ was 7.8 pM and 26 pM, respectively. (*S*/*N* = 3).

**Figure 4:**
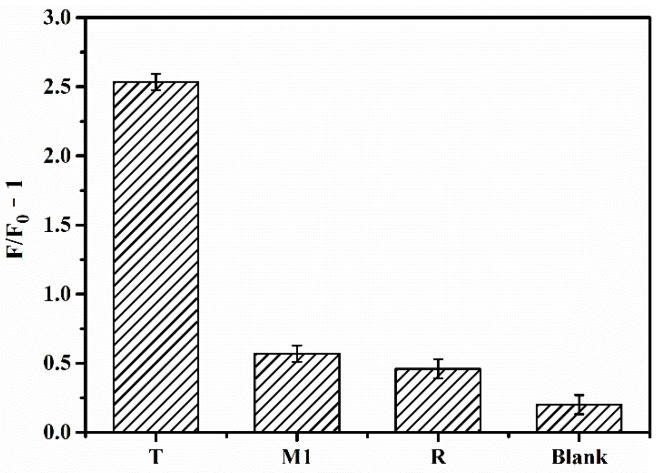
Selectivity of the biosensor. M1, single-base mismatch miRNA; R, noncomplementary random miRNA; T, target miRNA let-7a.

In order to assess the selectivity of the sensor, we compared the fluorescence recovery intensity of let-7a (T) with single-base mismatch miRNA (M1), noncomplementary random miRNA (R), and blank control under the same experimental conditions (Figure 4). (*F−F_0_*)/*F_0_* is the relative fluorescence intensity (the ratio of the fluorescence change value to the initial fluorescence value); (*F−F_0_*)/*F_0_* can be simplified to *F*/*F_0_* −1, where *F_0_* and *F* represent the initial fluorescence value at which the fluorescence was completely quenched by GO and the fluorescence recovery value after adding different microRNA, respectively. The values of *F*/*F_0_*−1 of T, M1, R, and Blank are 2.53, 0.57, 0.45, and 0.20, respectively, indicating that the fluorescence recovery rate of let-7a (T) was significantly higher than that of M1, R, and the blank control. Thus, even a single base mismatch markedly reduced fluorescence recovery, underscoring the high selectivity of the sensor.

Table 2 compares our miRNA detection strategy with other reported methods. Ma et al. nicely applied the distance-dependent photoinduced electron transfer of CuNPs-based biosensor for microRNA detection. Although this method was label-free, it had a relatively high detection limit due to lacking a signal amplification technique. Wu et al. designed 2D MnO_2_ nanoflakes, which exhibited a superior response to ssDNA over double-stranded DNA in the aspects of binding and catalytic to detect microRNA by electrochemical methods, though the process involved a series of complex material synthesis processes. Zou et al. developed a multiplex and fast detection platform for microRNAs based on a self-priming microfluidic chip and duplex-specific nuclease. This chip exhibited outstanding quantitative performance and specificity but required complicated chip preparation and time-consuming detection. There was a complex 3D DNA origami structure that enabled electrochemical detection of lung cancer-related microRNAs, which was sophisticated but lacked signal amplification technology. The biosensor developed in this study has a broader range of detection and a lower LOD without an enzyme or complicated operations.

### 3.4. Detection of miRNA in Serum by the Biosensor

In order to evaluate the clinical applicability of the DNA-based biosensor, we tested its performance in two clinical serum samples that did not contain the target miRNA let-7a. The samples were diluted with PBS and then mixed with a standard solution of let-7a, and fluorescence recovery was analyzed. The mean recovery rates for the two samples ranged from 90.80% to 97.03% (Table 3), confirming that the sensor can detect let-7a in human serum within a given range (0.01–1 nM).

## 4. Conclusions

We have established a nonenzymatic “on–off–on” fluorescence detection strategy based on nanomaterials for the sensitive and selective detection of a target miRNA. The excellent fluorescence quenching ability of GO significantly reduced the background signal for improved sensitivity, and the two TSDRs triggered by the target miRNA further enhanced fluorescence recovery. Based on its favorable performance, our novel biosensor has broad clinical applicability in disease diagnosis and monitoring and can potentially be adapted for the detection of other target biomolecules.

## Figures and Tables

**Table 1:** Nucleic acid sequences used in this study.

Name	Sequences (5′ to 3′)
Recognition DNA	(SH)- AAAAAAAAAAACTATACAACCTACTACCTCATAGGTAC
Hairpin DNA	ACAACCTATGAGGTAGTAGGTTGT
Fuel DNA	NH_2_-G*T*A*CCTATGAGGTAGTAGGT*T*G*
MicroRNA let-7a	UGAGGUAGUAGGUUGUAUAGUU
Non-recognition sequences	(SH)- AAAAAAAAACCGATCACAACGTACTACCTCAAAGGTTG
One base mismatch microRNA	UGAGGUAGUAGGUUGUCUAGUU

(* Phosphorothioate bonds. C: mismatched base in the sequence.).

**Table 2:** Comparison with previously developed sensors for miRNA detection.

Methodology	Analyte	Linear Range	LOD	Refs
Distance-dependent photoinduced electron transfer of DNA/Cu nanoparticles	miRNA let-7a	0.5 nM–100 nM	0.2 nM	[8]
Target-induced anti-shielding against the catalytic activity of two-dimensional nanozyme	miRNA let-7a	0.4 nM–140 nM	0.25 nM	[22]
Self-priming microfluidic chip	miRNA 100	100 pM–10 nM	45.35 pM	[23]
3D DNA origami nanostructures	miRNA	100 pM–1 μM	10.0 pM	[56]
Toehold-mediated nonenzymatic amplification on CDs and GO	miRNA let-7a	10 pM–1 nM	7.8 pM	This study

**Table 3:** Determination of target microRNA let-7a in clinical serum specimens.

Sample Number	Added (nM)	Founded (nM)	Recovery (%)	RSD (%) (*n* = 3)
1	0.01	0.00931	93.10	6.3
	0.50	0.47110	94.22	5.1
	1.00	0.97030	97.03	3.9
2	0.01	0.00908	90.80	4.7
	0.50	0.481010	96.20	4.6
	1.00	0.94120	94.12	3.2

Recovery (%) = 100 (concentration found/concentration added).

## Data Availability

Data sharing is not applicable to this article.

## Supplementary Materials

The following are available online at https://www.mdpi.com/article/10.3390/nano11102608/s1, Figure S1: Characteristics of CDs. (a) TEM of CDs. (b) CDs were excited at the green light and photographed directly. (c) Fluorescence spectra of CDs. (d) FT-IR spectra of CDs; Figure S2: Optical images of CDs illuminated under (a) white and (b) blue light; Figure S3: Black and white images of CDs. CDs were illuminated by (a) white light, (b) UV light and (c) blue light; Figure S4: (a) UV-vis absorption spectrum [64], (b) Excitation spectrum, (c,d) Excitation-emission matrices of CDs; Figure S5: Emission spectra of CDs at different excitation wavelengths; Figure S6: Effects of (a) NaCl (b) KCl concentrations, (c) pH on the fluorescence value of CDs. (d) photostability of CDs; Figure S7: Effects of (a) dsDNA-AuNPs concentrations and (b) fuel DNA-CDs concentrations on the *F/F_0_* −1 value of sensor system.

## Abbreviations

**Rank**	**Acronym**	**Common Meaning**
1	miRNA	microRNA
2	CDs	Carbon dots
3	GO	Graphene oxide
4	AuNPs	Gold nanoparticles
5	TSDRs	Toehold-mediated strand displacement reactions
6	FRET	Fluorescence resonance energy transfer
7	NHS	N-Hydroxysuccinimide
8	EDC	1-ethyl-3-(3-dimethylaminopropyl)carbodiimide
9	TCEP	Tris (2-carboxyethyl) phosphine hydrochloride
10	UV–vis	Ultraviolet–visible
11	FT-IR	Fourier transform infrared
12	HP	Hairpin
13	LOD	The limit of detection
14	LOQ	The limit of quantitation
