# Supplementary material for: Nonenzymatic DNA-Based Fluorescence Biosensor Combining Carbon Dots and Graphene Oxide with Target-Induced DNA Strand Displacement for microRNA Detection
Source: Nanomaterials (Basel). 2021 Oct 3;11(10):2608. doi: 10.3390/nano11102608 (PMC8537593; doi:10.3390/nano11102608)
Supplement: Supplementary file 1 [file nanomaterials-11-02608-s001.zip › nanomaterials-1386714-supplementary.pdf]

Supplementary Materials

Nonenzymatic DNA-Based Fluorescence Biosensor Combining Carbon Dots and Graphene Oxide with Target-Induced DNA Strand Displacement for microRNA Detection

Yuanyuan Gao [1,2], Hong Yu [1], Jingjing Tian [2,3] and Botao Xiao [1,4,*]

1 Guangdong Provincial Key Laboratory of Fermentation and Enzyme Engineering, School of Biology and Biological Engineering, South China University of Technology, Guangzhou 510006, China; 202010108508@mail.scut.edu.cn (Y.G.); 201920146464@mail.scut.edu.cn (H.Y.)

2 State Key Laboratory of Marine Resource Utilization in South China Sea, School of Material science and Engineering, Hainan University, Haikou 570228, China; tianjingjinghubei@163.com

3 Key Laboratory of Emergency and Trauma of Ministry of Education & Research Unit of Island Emergency Medicine of Chinese Academy of Medical Sciences, Hainan Medical University, Haikou 571199, China;

4 Joint International Research Laboratory of Synthetic Biology and Medicine, School of Biology and Biological Engineering, South China University of Technology, Guangzhou 510006, China;

* Correspondence: xiaob@scut.edu.cn; Tel.: +86-20-39380631

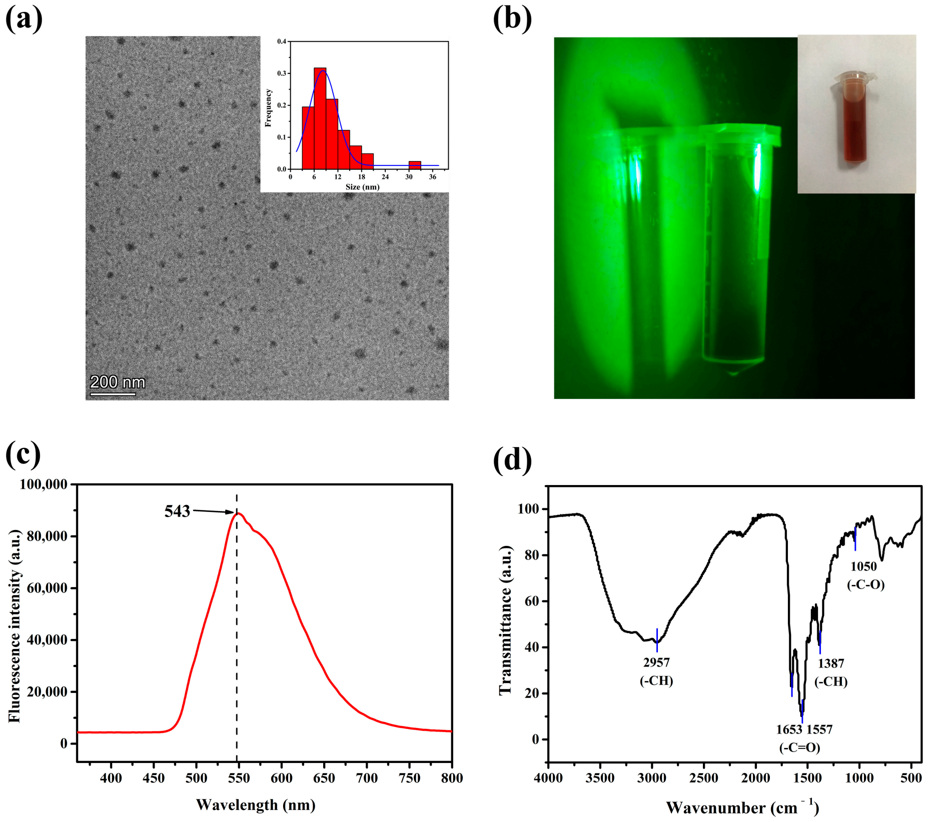

Figure S1. Characteristics of CDs. (**a**) TEM of image and size distribution (inset) of CDs. (**b**) CDs were excited at the green light and photographed directly. The inset was image of CDs solution under natural light. (**c**) Fluorescence spectra of CDs (excitation: 488 nm, emission: 543 nm). (**d**) FT-IR spectra of CDs.

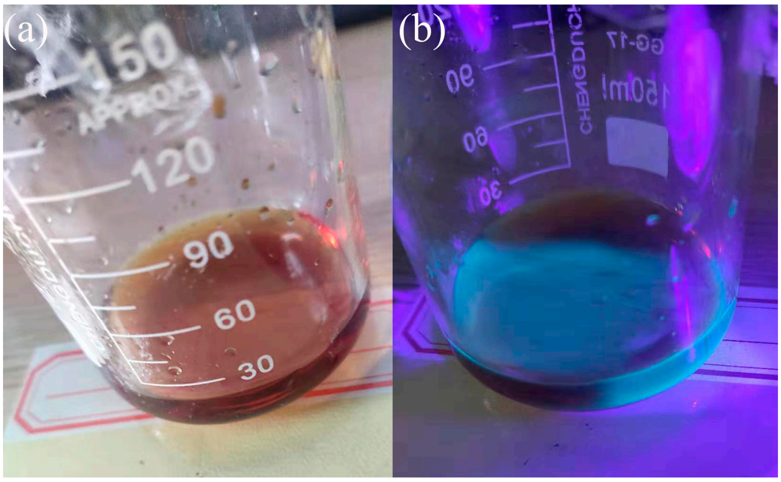

Figure S2. Optical images of CDs illuminated under (**a**) white and (**b**) blue light.

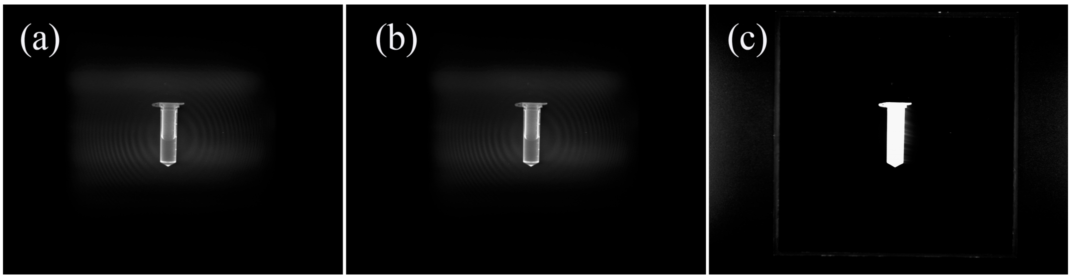

Figure S3. Black and white images of CDs. CDs were illuminated by (**a**) white light, (**b**) UV light and (**c**) blue light, then observed by a black and white camera. The exposure time was 2000 ms.

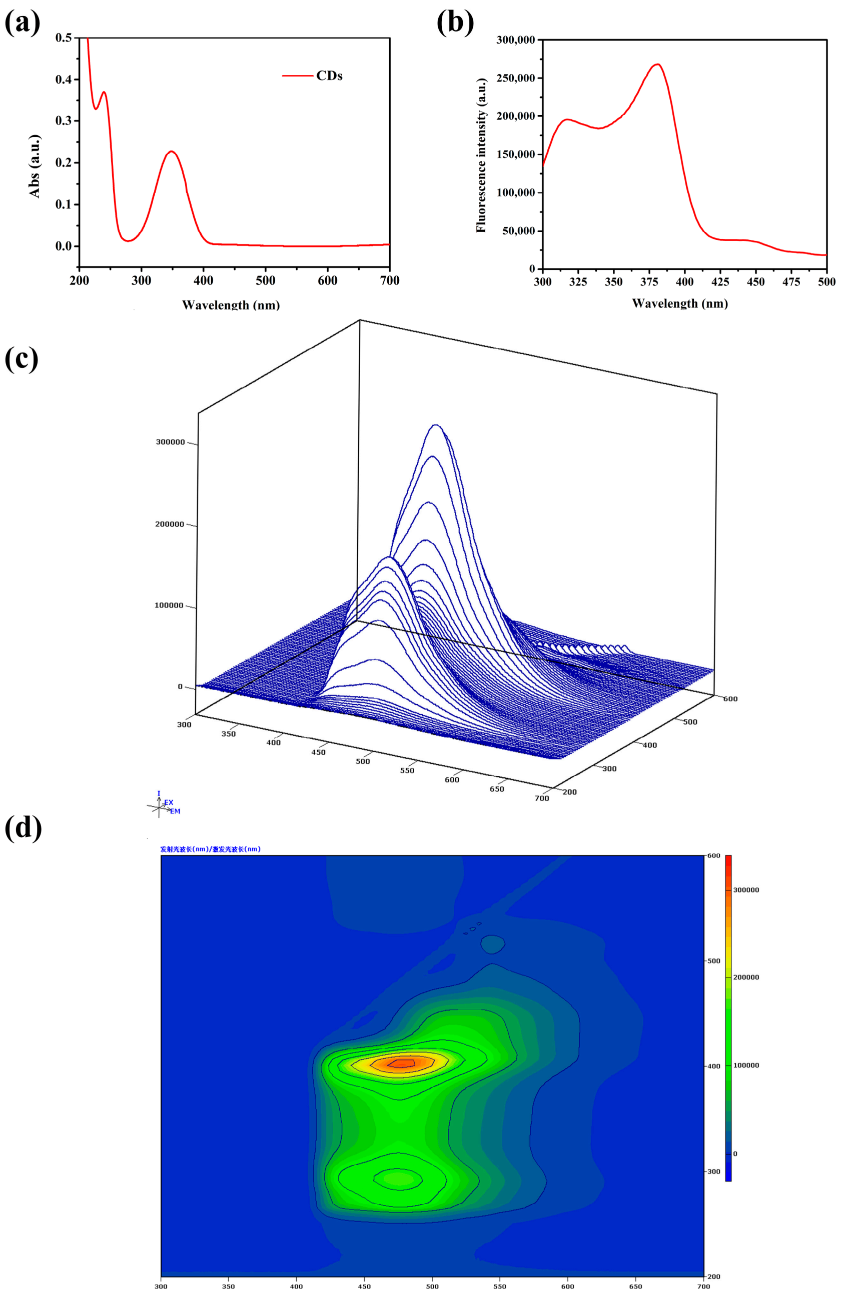

Figure S4. UV spectra of the CDs. (**a**) CDs have two absorption bands, located at 290 and 350 nm, which may be attributed to the

π-π* transition of C=C and the n-π* transition of C=O on the surface of CDs, respectively [1]. (**b**) Excitation spectrum, and (**c,d**) excitation-emission matrices of CDs. The fixed emission wavelength was 543 nm, and there were mainly three best excitation peaks for CDs. At ~488 nm, the difference in signals generated by different targets was sufficiently significant.

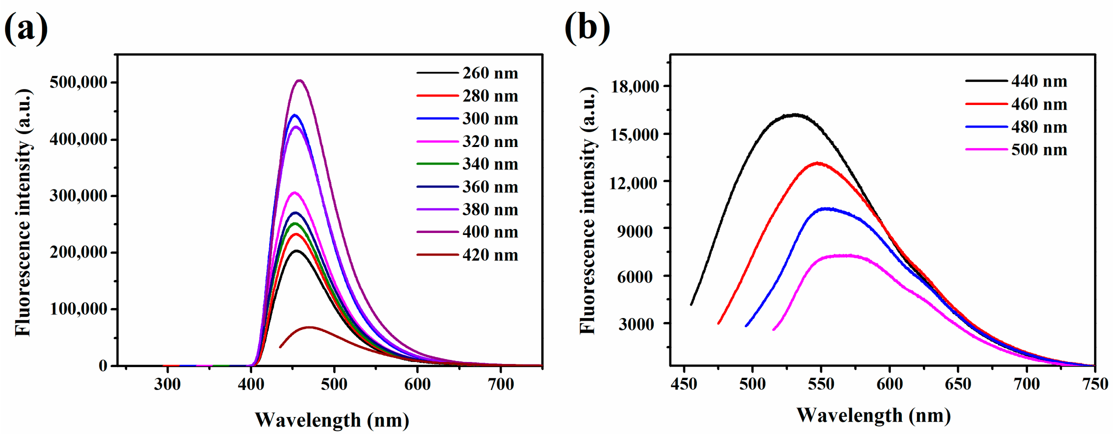

Figure S5. Emission spectra of CDs at different excitation wavelengths.

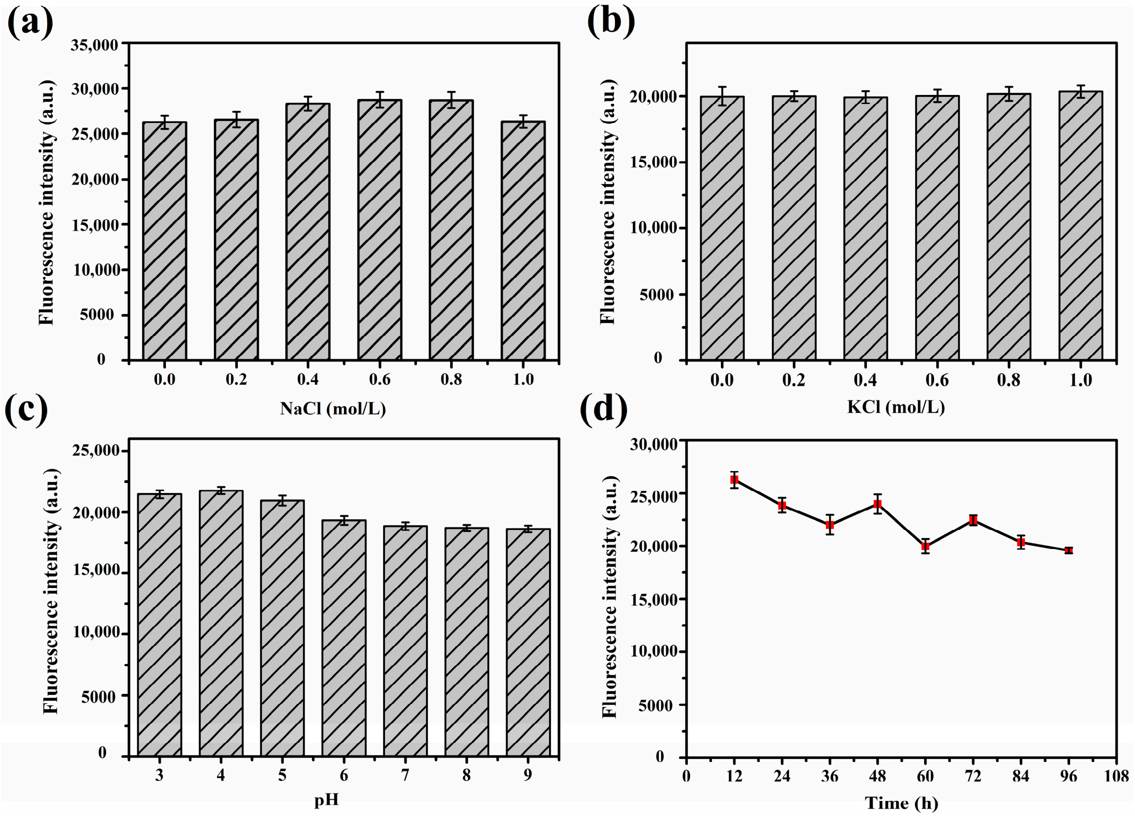

Figure S6. Effects of salt concentration and pH on the fluorescence of CDs. In the concentration range of 0-1 mol/L NaCl (**a**) and KCl (**b**), the fluorescence intensities of CDs have little change, which is conducive to their practical application in physiological salt environments. The fluorescence of CDs has a slight decrease under alkaline conditions (**c**), which may be related to the amino functional groups on the surface or the dispersion of CDs. (**d**) The luminescence stability of CDs in 96 h under the condition of excitation light of 488 nm.

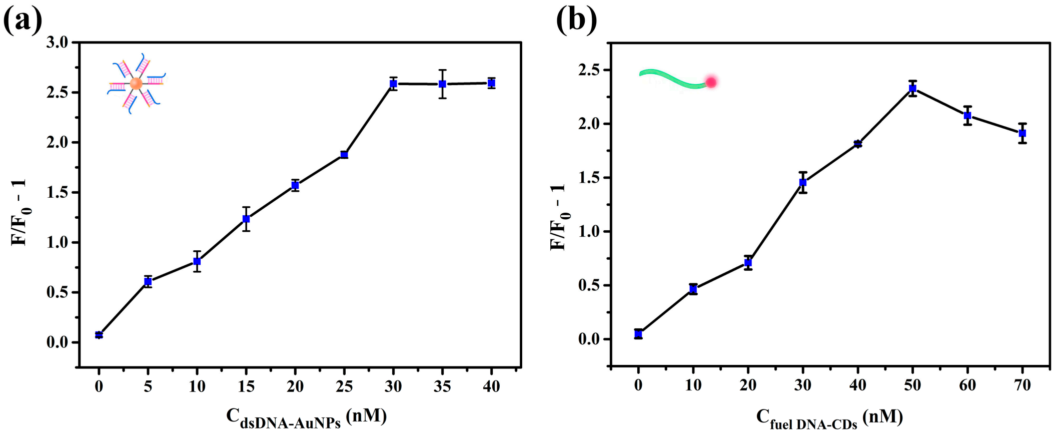

Figure S7. Effects of (**a**) dsDNA-AuNPs concentrations and (**b**) fuel DNA-CDs concentrations on the F/F_0–1 value of sensor system. When the concentration of dsDNA-AuNPs reaches 30 nM, the value of F/F_0–1 reached a plateau (**a**). The F/F_0–1 value showed a peak at 50 nM fuel DNA-CDs (**b**). Conditions: microRNA let-7a, 1 nM.

Reference

1. Kong, W.; Wu, H.; Ye, Z.; Li, R.; Xu, T.; Zhang, B. Optical properties of pH-sensitive carbon-dots with different modifications. *J. Lumin.* **2014**, *148*, 238-242.
